# Level of Theory and Solvent Effects on DASA Absorption Properties Prediction: Comparing TD-DFT, CASPT2 and NEVPT2

**DOI:** 10.3390/ma10091025

**Published:** 2017-09-03

**Authors:** Cristina García-Iriepa, Marco Marazzi

**Affiliations:** 1 Laboratoire Modélisation et Simulation Multi Echelle, Université Paris-Est, MSME, UMR 8208 CNRS, UPEM, 5 bd Descartes, 77454 Marne-la-Vallée, France; 2 Théorie-Modélisation-Simulation, Université de Lorraine—Nancy, SRSMC Boulevard des Aiguillettes, 54506 Vandoeuvre-lès-Nancy, France; 3 Théorie-Modélisation-Simulation, CNRS, SRSMC Boulevard des Aiguillettes, 54506 Vandoeuvre-lès-Nancy, France

**Keywords:** organic photoswitch, photochromic molecule, electronic absorption, two-photon absorption, molecular dynamics, density functional theory, multiconfiguration quantum chemistry methods

## Abstract

Donor–acceptor Stenhouse adducts (DASAs) are a very recent class of organic photoswitches that combine excellent properties, such as color and polarity change, a large structural modification, and excellent fatigue resistance. Despite their potential applications in different fields, very few studies have focused on rationalizing their electronic structure properties. Here, by means of different state-of-the-art theoretical methods, including solvent and vibrational effects, we show that while time dependent-density functional theory (TD-DFT) can qualitatively describe DASAs’ excited states, multiconfigurational quantum chemistry methods along with dynamic electron correlation (CASPT2, NEVPT2) are required for a quantitative agreement with the experiment. This finding is reasoned based on the different charge transfer characteristics observed. Moreover, the TD-DFT computed two-photon absorption properties are reported and suggested to red-shift the absorption band, as required for biological applications.

## 1. Introduction

Molecular switches are among the most studied molecular devices of the last few decades, mainly due to their versatility and remarkable applications [1,2]. Amid all of the possible external inputs needed to activate a switch, light has been widely used due to its clear advantages: easy and fast on/off switching, no generation of waste products, high spatial and temporal resolution, and an environmentally benign kind of energy [3,4]. Light-activated molecular switches are commonly known as photoswitches, and the research of novel systems and outstanding applications still attracts the interest of scientists. For this reason, this special issue is dedicated to efficient photoactive building blocks and their use in concrete applications.

Molecular photoswitches can be reversibly interconverted between two states with different molecular structures and properties. When the spectral properties (e.g., color) of these two states are clearly and easily distinguishable, these systems are considered photochromes. In the last decades, azobenzene, diarylethene and spiropyrans have been the more widely exploited photochromes [5,6,7]. Often, UV light is required to activate a colorless thermally stable state, limiting the applications in the biological and materials fields due to irreversible chemical damage and low penetration depth. Moreover, most azobenzene derivatives have an unnoticed color change between the two states, while some spiropyran and diarylethene compounds present low fatigue resistance [8,9,10,11,12].

**Figure 1:**
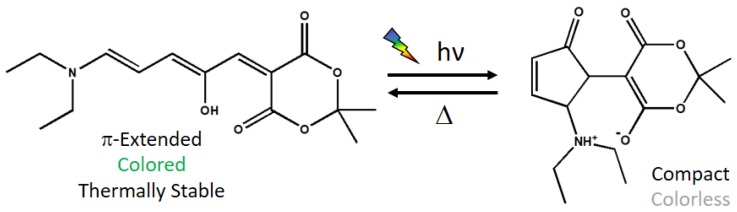
Switching mechanism of the donor–acceptor Stenhouse adducts (DASA) derivative under study. The opened state (**left**) is irradiated to form the closed state (**right**). Thermally, the closed state reverts to the opened state.

For these reasons, the discovery of the donor–acceptor Stenhouse adducts (DASAs) in 2014 [13,14] has shaken the field of photochromes as the thermally stable state is colored and hence, can be activated by visible light. In particular, a neutral opened π-extended colored form is converted by a 4π-Electrocyclization to a zwitterionic compact closed colorless form [13] (Figure 1) while passing through an intermediate generated by photoisomerization of one double bond [15]. The reverse reaction is thermally activated, as the compact form is not thermally stable.

It is remarkable that in only a few years, a considerable number of applications have been reported for DASA derivatives, including polymer science [16,17,18,19], targeted drug release [20,21], temperature localization [22], energy storage [23], the monitorization of photochemical transformations [24] and orthogonal photoswitching [18,25]. The breadth of applications is due to the versatility of this photochrome, as well as its outstanding properties: high molar absorptivity, photoswitching under visible light, and easy synthetic access [13,14]. Also, high fatigue resistance is observed, although some diarylethene derivatives register even higher values [26,27]. Moreover, it has been demonstrated that DASAs properties can be easily tuned by chemical modifications. In this regard, a first generation of DASAs have been reported based on dialkylamino donors [14]. In order to red-shift the maximum absorption wavelength and improve the solubility and photoswitching efficiency, a second generation of DASA derivatives was reported based on aromatic amine-based donors [28,29]. The absorption in the red or infrared region of the spectrum is especially required for biological applications. Apart from chemical modification, over the last few years the use of two-photon absorption (TPA) active compounds has emerged as a quite efficient and useful way to irradiate in the red and infrared windows [30,31,32,33,34]. In more detail, the molecule under irradiation simultaneously absorbs two photons, each one corresponding to half of the energy required by one-photon absorption (so called degenerate TPA). This way, the absorption wavelength is doubled. Moreover, since TPA is a non-linear process, the absorption probability is proportional to the square of the light source's intensity, hence increasing the spatial selectivity to the area of the laser beam focus. Indeed, TPA has found applications in medicine [35], laser scanning microscopy [36], optical data storage [37], bioimaging [38], etc. However, no studies have been reported up to now about the ability of DASA compounds for TPA. For this reason, one of the aims of this work is to compute the TPA cross-section and energy window of a given DASA derivative.

In order to propose novel DASA derivatives and related applications, it is crucial to know and investigate their properties. In this context, computational studies are quite relevant, as they allow researchers to analyze properties that are not experimentally accessible more in detail. However, just a few computational studies have been reported up to now. One of them focuses on the study of the different properties of the two isomeric states [39] by computing the excitation energies of two DASA derivatives at time dependent-density functional theory (TD-DFT) and Scaled Opposite Spin-Configuration Interaction with Single substitutions (SOS-CIS(D)) levels of theory. In both cases, a qualitative but not quantitative agreement with the experimental values has been achieved, finally suggesting a deeper study to be performed concerning the level of theory and solvent effects on the excitation energy. Apart from this fundamental work, coupled theoretical and experimental studies were performed to investigate structure–property relationships [15,28]. Concerning DASA applications, the only computational work describes titanium dioxide surfaces grafted with a DASA derivative [40].

Hence, a clear lack on the theoretical description of DASA derivatives is preventing the prediction of accurate properties. To possibly fill in this gap, the present computational work was performed with the following aims: (i) compute the excitation energies at different levels of theory including time-dependent density functional theory (TD-DFT) and more sophisticated multiconfigurational methods; (ii) study the effect of both implicit and explicit solvent on the excitation energy, and (iii) analyze DASA properties such as charge transfer character and TPA. This way, a protocol for the quantitative description of DASAs’ absorption properties is given, which moreover proposes TPA as a possible alternative solution to chemical modification, in order to consistently red-shift the absorption energy.

For this work, the thermally stable state of a prototypical DASA derivative has been selected. Indeed, it was studied both experimentally [13,15] and computationally [39]. It is composed by a diethylamino group as donor and a Meldrum’s acid based group as acceptor (Figure 1).

## 2. Results

Results are divided as follows: first, the effect of the level of theory on the vertical excitation energy and the nature of the electronic transition will be described. The TD-DFT, Complete Active Space 2^nd^ Order Perturbation Theory (CASPT2) and N-Electron Valence state 2^nd^ order Perturbation Theory (NEVPT2) methods will be compared, based on the gas phase optimized structure. In the following section, the solvent effect, both implicit and explicit, is introduced. The dynamics and vibrational effects will also be given, in order to obtain not only an estimated excitation energy corresponding to the absorption maximum, but also a spectral shape that can be better compared in the experiment. Moreover, solvent–chromophore interactions will be elucidated. To rationalize all findings, an accurate analysis of the electronic charges is then presented. Finally, the TPA intensity value is given, opening a discussion with respect to known TPA properties of existing photoswitches.

### 2.1. Level of Theory Effect

As can be seen in Table 1, the level of theory can strikingly affect the vertical transition to the lowest excited state. Indeed, while in all cases the S_0_–S_1_ transition corresponds to the optical brightest one compared with upper singlet states (see *f* values in Table 1), the S_0_–S_1_ energy is blue-shifted by all functionals and basis sets applied (i.e., TD-DFT, Table S2), when compared with the other methods.

In Table 1, we show the values related to the selected functional (B3LYP) and two different basis sets: 6-31+G(d), used to study the dynamical and solvent effects (see next sections), and cc-pVDZ, used to compare TD-DFT, CASPT2 and NEVPT2.

**Figure 2:**
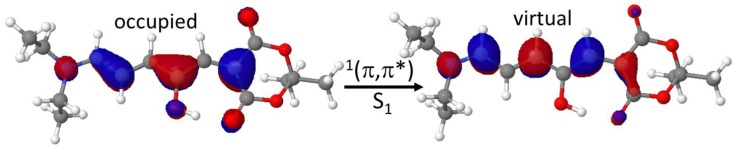
The occupied and virtual orbitals describing the electronic transition to the first excited state.

Especially, a red-shift of 0.32 eV (54 nm) and 0.41 eV (71 nm) is observed from B3LYP to the multiconfigurational methods NEVPT2 and CASPT2, respectively. When looking at the literature, an even larger red-shift can be found at the SOS-CIS(D) level, reaching 0.87 eV (130 nm) [39]. This latter corresponds to a sum-over-states formulation of the second-order perturbative double correction to configuration interactions with single substitutions. Since all TD-DFT values (see Table S2) give the same trend, irrespective of the functional and basis sets used, and that excited state perturbative theories are considered of a higher level than TD-DFT to describe excited states, we should conclude that DASA optical properties cannot be quantitatively assessed by TD-DFT, even though an analysis of the orbitals mainly involved in the S_0_–S_1_ vertical transition give the same overall picture at all levels (Figure 2): a ^1^(π,π*) excitation delocalized on the π-conjugated system. A partial rupture of the C=C double bonds character can be envisaged, as a first promoting cause of the photoisomerization step required to reach the intermediate reaction.

### 2.2. Solvent Effect

In this study, we have also investigated the effect of the solvent on the excitation energy of the selected DASA derivative. This way, we can discern the role of chromophore–solvent interactions on the absorption spectrum, and clarify their importance to achieve a fair agreement between the computed and the experimental value.

#### 2.2.1. Implicit Solvent

At first, the excitation energy has been computed considering implicit solvent by the polarizable continuum model (PCM) [41]. Three different solvents have been selected for analysis: (i) methanol, a polar and protic solvent; (ii) acetonitrile, a polar and aprotic solvent, and (iii) toluene, a non-polar solvent. The solvent effect has been investigated both at the TD-DFT and at the CASPT2 levels of theory, with the latter selected among the proposed perturbative multiconfigurational methods. By analyzing Table 2, it is found that for both levels of theory, the absorption wavelength is red-shifted by ca. 20 nm for methanol and acetonitrile, and ca. 30 nm for toluene, compared with the gas phase values of their respective level of theory (Table 1). Moreover, we can state that the absorption wavelength is shifted to the red if the polarity of the solvent decreases (e.g., from methanol to toluene), as experimentally observed. However, although this experimental trend can be qualitatively reproduced by TD-DFT calculations, the absolute values of the excitation energy are still quite blue-shifted compared with the experimental ones. Whereas, when using multiconfigurational methods such as CASPT2, the excitation energies computed considering implicit solvent reproduce the experimental trend both qualitatively and quantitatively. In particular, the absorption wavelengths computed at the CASPT2 level are in quite good agreement with the experimental data, with the largest difference found of 0.07 eV with toluene as solvent.

**Figure 3:**
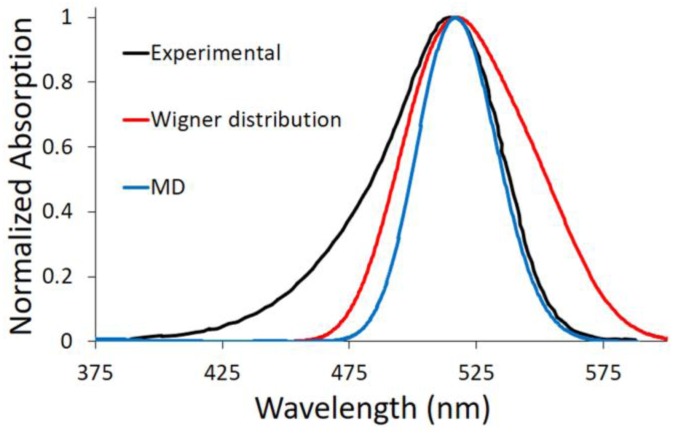
Comparison between simulated—by Wigner distribution and molecular dynamics (MD)—and experimental spectra (Adapted with permission from Helmy, S.; Oh, S.; Leibfarth, F.A.; Hawker, C.J.; Read de Alaniz, J. *J. Org. Chem.* **2014**, *79*, 11316–11329. Copyright 2017 American Chemical Society). Since time dependent-density functional theory (TD-DFT) is used, a red shift of 0.31 (0.48) eV is applied to the simulated spectra by Wigner distribution in order to better compare their shape with the experiment.

This procedure describes the implicit solvent effect on the excitation energy from a static point of view, hence lacking the vibrational effects. To consider both solvent and vibrational effects, we have performed a Wigner distribution based on the harmonic vibrational frequencies calculated for the ground state equilibrium structure optimized at the DFT level of theory, including methanol by PCM. Afterwards, a statistical number of geometries have been extracted and their excitation energies computed at the TD-DFT level of theory, using again the PCM. By performing this phase space sampling, it is possible to simulate the absorption spectrum as a convolution of all of the vertical transitions (red line in Figure 3). For the DASA derivative proposed here, the convergence of the simulated absorption spectrum was reached by considering 40 geometries (Figure S2A). By analyzing the simulated spectrum, the maximum absorption wavelength is found at 2.71 eV (457 nm) to be almost matching the value computed for the Franck–Condon geometry, i.e., the optimized ground state geometry in methanol (Table 2): 2.72 eV (455 nm). Taking into account that the Franck–Condon geometry is mainly planar, this means that no out-of-plane low-frequency high-amplitude mode contributes strikingly to the vibrational description. Instead, in-plane high-frequency modes and in-plane couplings of many low-frequency modes are implied.

#### 2.2.2. Explicit Solvent

The solvent has been also explicitly considered. In particular, a box of methanol molecules has been placed around the DASA derivative, applying periodic boundary conditions. Indeed, as a polar and protic solvent, methanol can interact to a large extent with the chromophore, especially by hydrogen bond interactions. A molecular dynamics (MD) trajectory of 10 ns has been performed and a statistical number of snapshots has been extracted (see Section 4 for details). Then, the excitation energy of each snapshot has been calculated at the B3LYP/AMBER level of theory, including the DASA chromophore in the quantum mechanics (QM) region. Finally, the absorption spectrum has been simulated by convolution of all the vertical transitions (blue line in Figure 3). In this case, the maximum absorption wavelength found is 2.88 eV (431 nm). This value is closer to the one computed in the gas phase (435 nm) than to the one calculated considering the solvent implicitly (455 nm).

In Figure 3, the two proposed computational approaches to include vibrational effects—i.e., Wigner distribution and MD—are compared to available experimental data [14]. Since we have demonstrated (see above) that TD-DFT describes only qualitatively the optical properties of this DASA derivative, an energy shift was applied to the computed spectra in order to match the experimental maximum absorption wavelength, and hence compare the spectra shape. As we can see, both MD and Wigner distribution methodologies fail in reproducing the band asymmetry (broadening) toward shorter wavelengths. We should note that the spectra simulations were performed using either three excited states (S_1_, S_2_ and S_3_) or only S_1_, resulting in almost indistinguishable spectra. Therefore, we can clearly state that only the lowest excited singlet state is involved in the spectroscopic signature. On the other hand, both of our approaches do not take into account the fine vibronic structure, which should be included in order to properly reproduce the high-energy part of the spectrum. Nevertheless, the low-energy spectrum side is usually considered the most relevant for application purposes, since it allows DASA derivatives to be applied with low-energy and hence more convenient energy inputs. In this respect, the MD approach presented here can finely reproduce the band shape toward longer wavelengths.

**Figure 4:**
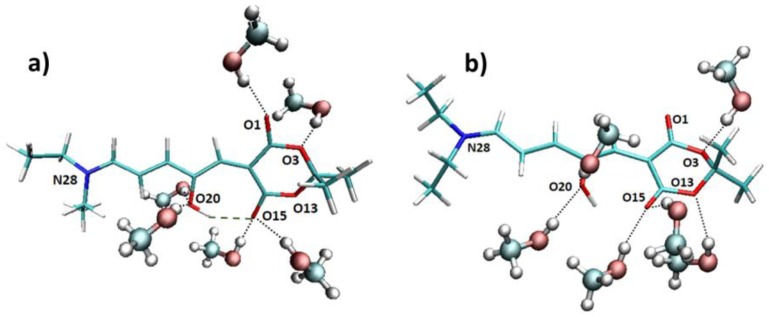
Two representative snapshots from the MD trajectory, depicting the two main hydrogen bonding patterns, which are characterized by (**a**) the formation and (**b**) the release of the intramolecular O20–H···O15 hydrogen bond.

Apart from the explicit solvent effect on the absorption wavelength, it is quite interesting to analyze the interactions between the methanol molecules and the chromophore along the trajectory. Looking at the molecular structure of the DASA derivative under study (Figure 1), we expect that an intramolecular hydrogen bond can be formed between the hydroxyl group and one oxygen atom of the six-membered ring. Indeed, when considering the Franck–Condon geometry in vacuum, the H···O15 distance is 1.65 Å, and the O20–H···O15 angle is 165 degrees (see Figure 4 for atoms numbering). However, it is possible that this hydrogen bond is disrupted by the interaction with a protic solvent such as methanol, in particular being replaced by hydrogen bonds with the methanol hydroxyl group. More in general, N28, O1, O3, O13, O15, and O20 could all in principle interact with methanol molecules. With this purpose, we have analyzed the hydrogen bond patterns found along the trajectory in terms of the average number of hydrogen bonds between the solvent molecules and the chromophore (Table S3). It is observed that N28 almost does not interact with methanol, O3 and O13 are bound to one methanol molecule for half of the simulation time, O1 stably forms one hydrogen bond, whereas O15 and O20 (i.e., the oxygen atoms expected to establish the intramolecular interaction) form one to two hydrogen bonds with methanol. Such interactions with methanol result in the weakening of the intramolecular O20–H···O15 hydrogen bond, finally resulting in an almost free rotation of the O20–H hydroxyl group. Indeed, two main patterns are found along the trajectory, respectively with (Figure 4a) or without (Figure 4b) an intramolecular hydrogen bond formed.

We can hereby conclude that protic solvents could interact to a large extent with DASA derivatives, and that an explicit description of these interactions gives a more complete picture of the system under study. However, as shown in Figure 3, the absorption wavelength computed by the MD approach is substantially blue-shifted compared with the experimental value (0.48 eV). Hence, the electronic description of the excited state at the TD-DFT level is suitable only for a qualitative description. Perturbative multiconfigurational quantum chemistry methods are necessary for a quantitative description.

### 2.3. Analysis of the Electronic Charges

**Figure 5:**
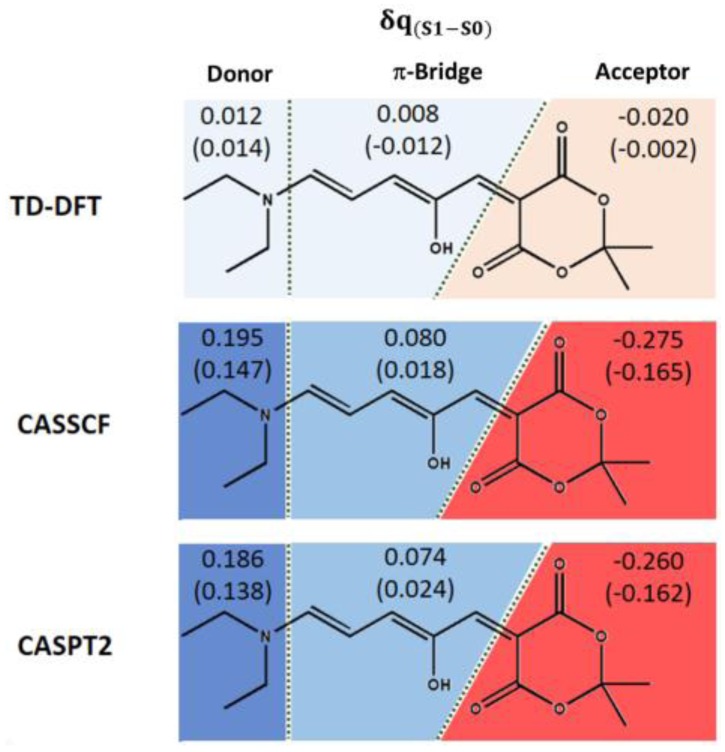
Mulliken charge analysis at TD-DFT (B3LYP and CAM-B3LYP), CASSCF, and CASPT2 levels of theory. The photoswitch is divided into three groups of atoms: donor, π-bridge, and acceptor. For each group, the charge difference between ground and the lowest singlet excited state, δq_(S1–S0)_, is given in vacuum, and in brackets when including methanol as solvent by the polarizable continuum model (PCM). The background color intensity is proportional to the charge value (blue: positive charge; red: negative charge).

The partial atomic charges were analyzed in order to investigate the origin of the differences in the predicted optical properties between TD-DFT and multiconfigurational quantum chemistry methods. Especially, both the Complete Active Space Self Consistent Field (CASSCF) wave function—used as reference for the perturbative treatment—and the CASPT2 calculation were analyzed, to address eventual variations when including only the static electron correlation (CASSCF) or also the dynamic component (CASPT2). As shown in the Supplementary Materials, when compared to natural bond orbital (NBO) charges, Mulliken charges can be considered reliable when adding up the charges of groups of atoms. In this case, the chromophore was split into donor, π-bridge, and acceptor moieties. Figure 5 shows that the TD-DFT description results in a very limited (almost null) charge transfer character, giving the same values with the hybrid B3LYP and the range-corrected CAM-B3LYP functional, the latter being usually applied to describe charge transfer states. On the other hand, both CASSCF and CASPT2 calculations result in a better description of the partial charge transfer character (ca. 0.2–0.3). This can possibly explain the necessity to include a multiconfigurational description to quantitatively predict DASAs’ properties. Especially if even at a lower extent, the π-bridge always participates as a moderate donor and lets only the six-membered ring as acceptor. The trend is confirmed when considering methanol by PCM (Figure 5, values in brackets), although this model slightly lowers the charge transfer character.

### 2.4. Two-Photon Absorption Properties

The TPA calculated absorption intensity of the DASA derivative in vacuum is 3.32 GM, a non-negligible value when compared with TPA values of photoswitches already applied in different fields, e.g., retinal isomerization in the Rhodopsin protein to trigger human infrared vision (TPA cross section of ca. 2 GM) [42]. This demonstrates that larger TPA cross-section values are welcome, but not strictly required for two-photon activation, since the energy red-shift—compared with one-photon absorption—is the most important parameter for application purposes. Indeed, biological and medical applications could especially take advantage of TPA, since water is usually the solvent of choice and it is expected to exhibit behaviors similar to methanol (both protic and polar solvents). Especially, the simultaneous absorption of two photons would reduce the required irradiation energy by a factor of two, resulting in 1.20 eV (1030 nm) in methanol, i.e., falling in the near-infrared therapeutic window (from 650 to 1350 nm) where the penetration of biological tissues is maximal [14]. Certainly, chemical modifications may be envisaged to increase the TPA cross-section of a chromophore once its TPA activity is established.

## 3. Discussion

A static and dynamic study of a prototypical DASA derivative was performed in gas phase, including various solvents and using different methodologies and levels of theory. We have shown that TD-DFT describes the absorption spectrum only qualitatively, since blue-shifted vertical transition energies are found, as also mentioned in previous studies [39]. On the other hand, perturbative multiconfigurational methods (such as CASPT2 and NEVPT2) do reproduce the DASA absorption properties almost quantitatively. While NEVPT2 can substantially reduce the blue-shift, the CASPT2 method matches well with the experiment, but only when properly selecting the Ionization Potential Electron Affinity (IPEA) shift (see Supplementary Materials). If we follow the suggestion of Zobel et al.—i.e., discard the IPEA shift to calculate CASPT2 excited states of organic chromophores [43]—a red-shift is found when compared with the experiment, which agrees with previous perturbative configuration interaction calculations [39].

The inclusion of solvent—by both implicit and, for the first time in DASA studies, explicit models—does not explain the quantitative disagreement found between TD-DFT and experimental values. Nevertheless, both TD-DFT and CASPT2 methods result in the same solvent-induced red-shift (0.1 to 0.2 eV), when compared with the respective excitation energy calculated in the gas phase. We can hence conclude that solvent effects are equally treated at both levels of theory.

In order to explain the discrepancy between TD-DFT and multiconfigurational methods, a charge analysis was conducted which elucidated that a partial charge transfer character from the ground to the first excited state can be observed only if at least the CASSCF level is used. On the other hand, the inclusion of the dynamic electron correlation by the CASPT2 method does not modify the type and extent of charge transfer.

Concerning the absorption band shape, we found that the MD proposed approach to sample the electronic ground state results in a fine description of the spectrum toward lower energies (i.e., longer wavelengths), while the tail experimentally recorded toward higher energies (i.e., shorter wavelengths) cannot be properly reproduced with a PCM-coupled Wigner distribution approach. We should therefore rely on a higher-level vibronic description, as previously shown [39], if we are interested in modeling the overall absorption spectrum shape.

Nonetheless, the interest in the application of DASA derivatives is usually devoted to the absorption in the red and possibly infrared energy window. With this aim, TPA properties were calculated in gas phase, and an encouraging absorption intensity of ca. 3 GM was found. When compared with other commonly used photoswitches, the value found for this DASA derivative is larger (0.9 GM for azobenzene [44]) or of the same order of magnitude (8 GM for stilbene [45]). Moreover, it has been shown that for both azobenzene and stilbene, the TPA absorption intensity can be easily enhanced by modification of the electronic structure (e.g., introducing novel donor and/or acceptor groups, or increasing the conjugation extent) [44,46]. Indeed, we envisage in the near future the design of novel DASA derivatives with enhanced TPA intensity by applying the strategy proposed here. Moreover, it should be pointed out that the TPA band experimentally measured for azobenzene and stilbene is located at wavelengths shorter than 500 nm, and hence out of the visible spectral window required for biological applications. In comparison, the two-photon excitation of DASA derivatives could be performed with red or even infrared light, which opens a wide variety of biological applications. We should note that DASA derivatives are not the only open–closed photoswitch family proposed to be activated by one- and two-photon absorption. Diarylethenes were also found to be highly efficient, moreover offering high TPA cross-sections, although only when chemically modified to the degree of 40 to 70 GM for opened structures [47,48] and several hundred GM for closed structures [48]. Nevertheless, the change in the end-to-end distance offered by diarylethenes is very limited compared with DASAs.

## 4. Materials and Methods

The selected photoswitch was first optimized on the ground state in vacuum at the B3LYP/6-31+G(d) level, followed by a calculation of the vertical excitation energies at different levels of theory. Especially, time dependent-density functional theory (TD-DFT) was applied by benchmarking different functionals and basis sets (see Supplementary Materials), increasing the benchmark set that Laurent et al. performed on the same system [39]. Considering that almost no difference was found among them, the B3LYP functional [49,50] method was selected coupled to the cc-pVDZ basis set for comparison with multiconfigurational quantum chemistry methods: CASPT2/cc-pVDZ [51] and strongly-contracted NEVPT2/cc-pVDZ [52], in both cases selecting an active space of 14 electrons in 13 orbitals (see details in Supplementary Materials). The same states when including implicit solvent were analyzed by the polarizable continuum model (PCM) [41]. In this case, different solvents (methanol, acetonitrile and toluene) were tested by optimizing the ground state structure for each solvent at the B3LYP/6-31+G(d) level, followed by TD-DFT excited state calculations. A direct comparison with the CASPT2 method was then performed.

To rationalize the charge transfer character of the DASA model, a Mulliken population analysis was performed at TD-DFT, CASSCF (i.e., the reference wave function for CASPT2 and NEVPT2 calculations), and CASPT2 levels. Moreover, natural bond orbital (NBO) analysis was performed at the TD-DFT level to validate the limits of the Mulliken analysis. Since charge analysis is crucial to investigate the nature of the vertical transition, the hybrid B3LYP and the range-separated CAM-B3LYP [53] functionals were both used for TD-DFT analysis. No difference was evident between them, which validated once again the choice of B3LYP for all TD-DFT calculations.

The TPA cross-section was hence calculated at the B3LYP/6-31+G(d) level for the structure optimized on the ground state in vacuum by applying the quadratic response of the single residue formalism.

Also, the B3LYP functional with the 6-31+G(d) basis set was employed to investigate the vibrational and dynamical effects. This was done by a preliminary exploration of the ground state conformational space, followed by the excited state calculation of an ensemble of snapshots. Finally, the absorption spectrum was obtained as a convolution of all vertical transitions (full-width at half-maximum = 0.2 eV). Here, we proposed two different strategies concerning the conformational search: (i) an exploration of the vibrational normal modes by performing a Wigner distribution based on the harmonic vibrational frequencies calculated at the ground state equilibrium geometry, followed by vertical transition energy calculations of the generated structures; (ii) perform classical molecular dynamics (MD), followed by vertical transition energy calculations of randomly selected structures along the trajectory, using hybrid quantum mechanics/molecular mechanics (QM/MM). The QM region corresponds to the photoswitch, while all methanol molecules compose the surrounding MM region. As already conducted elsewhere by some of the authors, comparison of the two approaches gives valuable structure and spectroscopic insights [31,33,34]. The latter strategy requires the accurate parameterization of the photoswitch force field. Especially, charges have been obtained by the standard Restrained Electro-Static Potential (RESP) procedure [54], while all of the parameters are based on the generalized amber force field (gaff) [55]. Both the charges and the parameters have been refined by QM/MM calculations, as explained below. As for MD, the DASA model was surrounded by 480 methanol molecules (parameters of methanol taken from the solvent library available in AMBER 2016 program [56]) in an octahedral box and applying periodic boundary conditions. The system was firstly minimized (2000 steps with the steepest descent algorithm and 2000 steps with the conjugated gradient algorithm), followed by thermalization and equilibration before an MD production simulation was run in the isothermal-isobaric (NPT) ensemble for 10 ns at constant temperature (300 K) and constant pressure (1 atm), as assured by the Berendsen algorithm [57]. The last snapshot of the MD trajectory has been extracted and optimized at the B3LYP/AMBER level of theory using the 6-31+G(d) basis set. The charges of the optimized structure have been calculated with the NBO method. This way, the parameters and charges of the switch have been refined and once updated, a novel MD production trajectory of 10 ns has been performed. Two refinement steps were necessary to reach convergence of the convoluted absorption spectrum.

Concerning software, all TD-DFT calculations were performed with Gaussian09 (Gaussian Inc., Wallingford, CT, USA) [58] except for the TPA calculation, which was conducted with Dalton2016 (University of Oslo, Oslo, Norway) [59]. For B3LYP/AMBER optimizations, a Gaussian09/Tinker (Washington University, Saint Louis, Missouri, USA) [60] protocol was applied, based on electrostatic embedding. CASPT2 and NEVPT2 computations were performed with the Molcas8 (Lund University, Lund, Sweden) [61] and Orca3 (Max-Planck-Gesellschaft, Mülheim an der Ruhr, Germany) [62] suite of programs, respectively. The Wigner distribution was realized with the Newton-X program (University of Vienna, Vienna, Austria and University of Pisa, Pisa, Italy) [63], while MD was performed with the Amber 2016 software (University of California, San Francisco, California, USA) [56].

## Figures and Tables

**Table 1:** Vertical excitation energies (*E*_S0→S1_) and oscillator strengths (*f*) at different levels of theory and basis sets, including data from the literature.

Level of Theory	E_S0→S1_ eV (nm)	*f*
B3LYP/6-31+G(d)	2.85 (435)	1.21
B3LYP/cc-pVDZ	2.88 (431)	1.20
NEVPT2/cc-pVDZ	2.56 (485)	1.02
CASPT2/cc-pVDZ	2.47 (502)	1.48
SOS-CIS(D)/SBS ^1^	2.21 (561)	

^1^ Data taken from reference [39].

**Table 2:** Vertical excitation energies (*E*_S0→S1_) for different solvents. All simulated values are calculated by the polarizable continuum model (PCM). All values are given in eV (nm).

Solvent	Experiment	*E*_S0→S1_ TD-DFT	$\Delta E_{PCM-Gas}^{TD-DFT}$	*E*_S0→S1_ CASPT2	$\Delta E_{PCM-Gas}^{CASPT2}$
Methanol	2.41 (515) ^1^	2.72 (455)	0.14 (20)	2.38 (522)	0.09 (20)
Acetonitrile	2.36 (525) ^1^	2.72 (456)	0.13 (21)	2.37 (523)	0.10 (21)
Toluene	2.27 (545) ^1^	2.64 (469)	0.21 (34)	2.34 (530)	0.13 (28)

^1^ Data taken from reference [14].

## Supplementary Materials

The following are available online at www.mdpi.com/1996-1944/10/9/1025/s1: computational details, effect of different functionals and basis sets for TD-DFT calculations, theoretical spectra convergence, hydrogen bonds analysis, charge analysis and Cartesian coordinates.
