# Supplementary material for: Level of Theory and Solvent Effects on DASA Absorption Properties Prediction: Comparing TD-DFT, CASPT2 and NEVPT2
Source: Materials (Basel). 2017 Sep 3;10(9):1025. doi: 10.3390/ma10091025 (PMC5615680; doi:10.3390/ma10091025)
Supplement: Supplementary file 1 [file materials-10-01025-s001.pdf]

Supplementary Materials

Level of Theory and Solvent Effects on DASA Absorption Properties Prediction: Comparing TD-DFT, CASPT2 and NEVPT2

Cristina García-Iriepa[1,*] and Marco Marazzi[2,3,*]

[1] Université Paris-Est, Laboratoire Modélisation et Simulation Multi Echelle, MSME, UMR 8208 CNRS, UPEM, 5 bd Descartes, 77454 Marne-la-Vallée, France
[2] Théorie-Modélisation-Simulation, Université de Lorraine – Nancy, SRSMC Boulevard des Aiguillettes, Vandoeuvre-lès-Nancy, France
[3] Théorie-Modélisation-Simulation, CNRS, SRSMC Boulevard des Aiguillettes, Vandoeuvre-lès-Nancy, France

Content

1. Computational details
2. Effect of different functionals and basis sets for TD-DFT calculations
3. Theoretical spectra convergence
4. Hydrogen bonds analysis
5. Charge analysis
6. Cartesian coordinates

1. Computational details

Different multiconfigurational quantum chemistry methods were applied in this work: static electron correlation was taken into account by the Complete Active Space Self Consistent Field approach (CASSCF), by state averaging over the ground state (S_0) and the two lowest-lying excited singlet states (S_1 and S_2). An active space of 14 electrons-in-13 orbitals was found to correctly describe the system. Indeed, increasing the size of the active space does not result in different excitation energies, since the added virtual orbitals do not almost participate. Moreover, to include the dynamic electron correlation, required to obtain quantitative results to be compared with the experiment, the second-order perturbation-theory (PT2) was applied to the CASSCF wavefunction in two ways: CASPT2 (Complete Active Space PT2) and NEVPT2 (N-Electron Valence state PT2), in both cases applying the state-specific scheme.

While the NEVPT2 does not require any parameter, the CASPT2 calculations were performed with different imaginary shifts and IPEA values, in order to test their effect on the vertical transition energy (Table S1). As it can be seen, the imaginary shift does not play a significant role in the range 0.0 – 0.2, while the excitation energy is more sensitive to the choice of the IPEA value. Especially, keeping the default value in Molcas8 (0.25) the excitation energy is blue shifted compared to the experiment, while avoiding to include the IPEA correction (0.0) results in a red shifted absorption. Hence, only an adjusted IPEA value of 0.1 gives results in line with the experiment (515 nm), considering a red shift of around 20 nm ascribed to the solvent – water or methanol – as calculated by PCM (see table 2 in main text).

Table S1. Excitation energy values calculated at the CASPT2 level of theory using different imaginary shifts and IPEA values.

Imaginary Shift	IPEA value	Energy eV (nm)
**0.2**	0.25	2.64 (469)
**0.2**	0.1	2.47 (502)
**0.2**	0.0	2.30 (539)
**0.0**	0.1	2.45(507)

When including vibrational and dynamics effects through the Wigner distribution, a set of vibrational frequencies and the corresponding normal mode vectors were provided by a frequency calculation performed on the Franck−Condon geometry at the B3LYP/6-31+G(d) level of theory. Once generated all snapshots corresponding to the ground state sampling, TD-DFT calculations were performed by applying PCM to simulate the environment.

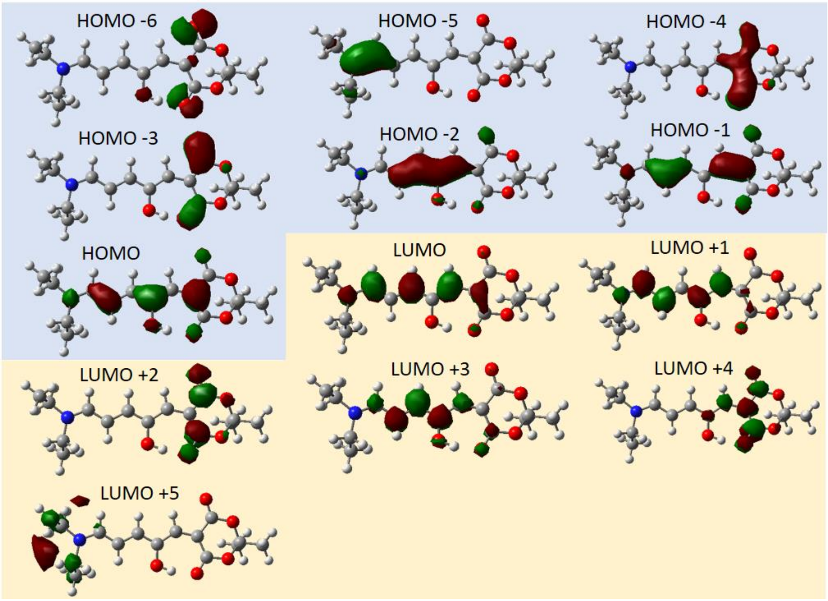

Figure S1. Active Space of 14 electrons-in-13 orbitals, *i.e.* CAS(14,13), selected for the multiconfigurational quantum chemical calculations (HOMO: Highest Occupied Molecular Orbital; LUMO: Lowest Unoccupied Molecular Orbital).

2. Effect of different functionals and basis sets for TD-DFT calculations

The excitation energy of the selected DASA derivative has been calculated at the TD-DFT level of theory. Different functionals and basis sets have been tested in order to check their effect on the excitation energy value. More in particular, nine different functionals, including hybrid, long-range-corrected and exchange functionals, have been tested keeping the 6-31+G(d) basis set. Moreover, five different basis sets have been tested for both the B3LYP and CAM-B3LYP functionals. By analyzing Table S2, we can conclude that the use of large basis sets such as Def2TZVP or cc-pVDZ almost keep invariant the value obtained with small basis sets such as 6-31+G(d). In addition, the effect of the functional was also found to be small. Indeed, the largest variation found, 435 nm for B3LYP and 427 nm for LC-BLYP, corresponds to only *ca.* 0.05 eV, hence lying well below the error admitted by the method. So, we can conclude that both the basis set and the functional selected for the calculation has almost no effect on the excitation energy. Also, the same excitation energy values have been obtained when empirical dispersion (GD3 and GD3BJ) have been considered in the calculation.

Table S2. S_0-S_1 excitation energy in eV (nm) of the structure optimized on the ground state, calculated at the TD-DFT level of theory using different basis sets and functionals.

Basis set / Functional	6-31+g(d)	6-311+g(2d,p)	cc-pVDZ	Aug-cc-pVDZ	cc-pVTZ	Aug-cc-pVTZ	Def2TZVP
**B3LYP**	2.85 (435)	2.83 (438)	2.88 (431)	2.83 (439)	2.86 (434)	2.83 (438)	2.85 (435)
**CAM-B3LYP**	2.88 (430)	2.87 (432)	2.89 (429)	2.86 (433)	2.89 (429)	2.86 (434)	2.88 (430)
**wB97xD**	2.87 (432)						
**B3P86**	2.86 (433)						
**B3PW91**	2.86 (434)						
**LC-BLYP**	2.90 (427)						
**LC-WPBE**	2.87 (432)						
**PW91**	2.89 (429)						
**B98**	2.87 (432)						

3. Theoretical spectra convergence

In this study, the absorption spectrum of the selected DASA derivative has been simulated following two procedures: *i)* generation of a Wigner distribution and calculation of the excitation energies for different structures along the sampling and *ii)* production of a MD simulation with explicit methanol molecules and calculation of the excitation energy for different snapshots along the trajectory. In both cases, it has been checked the convergence of the maximum absorption wavelength and of the shape of the absorption band, with the number of snapshots considered. By analyzing Figure S2A, we can conclude that 40 snapshots are enough to reach the convergence for the spectrum simulated using the Wigner distribution. Similarly, in Figure S2B we observe that again, 40 snapshots are enough to reach the convergence of the absorption spectrum shape for the MD approach.

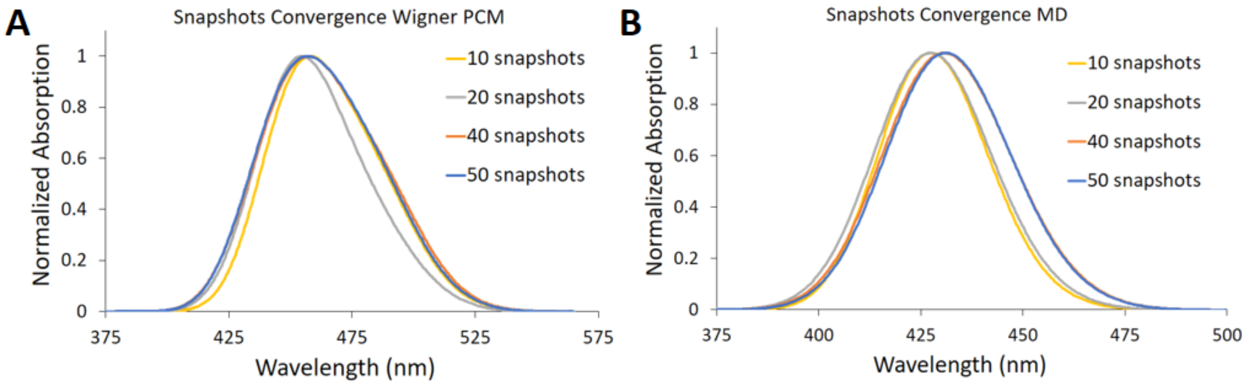

Figure S2. Absorption spectra calculated by convoluting an increasing number of structures, for convergence purposes, by applying a ground state sampling based on A) Wigner distribution and B) MD trajectory.

4. Hydrogen bonds analysis

The interaction between the solvent and the chromophore has been investigated. Methanol has been selected for this study as it is a polar solvent that can easily interact with the DASA derivative under study, as it has polar centers such as a hydroxyl group or other oxygen atoms. For this aim, the hydrogen bonds taking place between the methanol molecules and the chromophore along the trajectory have been analyzed. This way, the average fraction of hydrogen bonds as well as the average distance and angle between the two atoms involved in this interaction are obtained with the cpptraj tool of Amber program (Table S3).

Table S3. Hydrogen bond analysis along the MD trajectory (see Figure 4 in the main text for atoms labels).

Donor	Acceptor	Average Fraction of HB	Average Distance (Å)	Average Angle (deg)
**O15**	MeOH	1.6080	2.7180	162.0533
**O20**	MeOH	1.2960	2.7461	160.9224
**O1**	MeOH	1.0600	2.7510	160.6497
**O13**	MeOH	0.5880	2.7949	158.5703
**O3**	MeOH	0.5720	2.7861	159.4232
**N28**	MeOH	0.0300	2.9080	161.4343

5. Charge analysis

The atomic charges in the ground state of the DASA derivative under study have been computed with both the NBO and Mulliken population methods. In both cases, the atomic charges have been computed using the B3LYP functional and three different basis sets have been tested: 6-31+G(d), def2TZVP and cc-pVDZ. In order to present the results, the charge of the donor, π-bridge and acceptor groups (see Figure 5 in the main text for groups definition) have been calculated. Regarding to the Mulliken population method (Figure S3A), a clear dependence of the charges with the basis set has been observed: the charges computed with the 6-31+G(d) are different from the ones calculated with the larger basis sets def2TZVP and cc-pVDZ. Moreover, similar values have been obtained for the def2TZVP and cc-pVDZ basis sets. Whereas, for the charges computed with the NBO method, no dependence with the size of the basis set has been found (Figure S3B). Hence, we can conclude that the Mulliken charges computed with small basis sets such as 6-31+G(d) could be not valid and a large basis set is needed for the calculation.

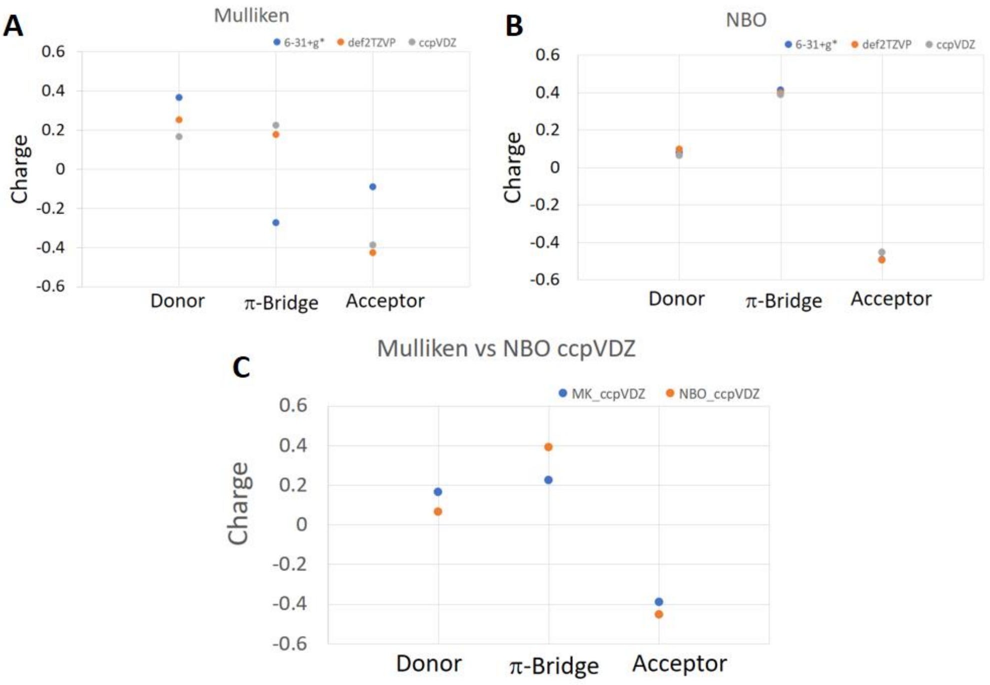

Figure S3. Charge dependence with the basis set computed with the A) Mulliken and B) NBO population methods. C) Comparison of the Mulliken and NBO charges computed with the cc-pVDZ basis set (see Figure 5 in the main text for donor, π-bridge and acceptor groups nomenclature).

As presented in the main text, the Mulliken charges of the DASA derivative computed at the TD-DFT, CASSCF and CASPT2 levels of theory have been compared. In this case, the cc-pVDZ basis set has been selected for the calculation. In order to check if the Mulliken charges computed with this basis set are valid, we have computed the NBO charges with the same basis set and compared the results. As observed in Figure S3C, the charges of the three different groups (donor, π-bridge and acceptor) are comparable. Hence, the Mulliken charges computed with this basis set are reliable.

6. Cartesian coordinates

Cartesian coordinates in Ångström of the ground state equilibrium structure optimized at the B3LYP/6-31+g(d) level of theory.

C	-5.006724	-0.121134	0.230819
C	-2.865191	-0.945954	-0.535079
C	-2.305140	0.341736	-0.150231
C	-3.215269	1.488427	0.032407
C	-6.416626	-0.212484	-0.327052
C	-4.926007	-0.452104	1.721900
C	-0.944448	0.632760	-0.024469
C	0.239742	-0.132081	-0.132087
C	1.452231	0.523058	0.079020
C	2.714575	-0.102145	-0.006488
C	3.878967	0.606386	0.210310
C	5.453199	-1.249333	-0.167519
C	5.384059	-2.170046	1.057028
C	6.275739	1.059803	0.382902
C	6.888035	1.615790	-0.907905
N	5.149300	0.150341	0.149056
O	-2.233747	-1.929852	-0.922894
O	-2.870070	2.646476	0.168200
O	-4.560930	1.214824	-0.009061
O	-4.219503	-1.064525	-0.521171
O	0.294959	-1.458908	-0.410482
H	-7.067732	0.494310	0.195427
H	-6.804936	-1.226604	-0.195437
H	-6.408055	0.031584	-1.392792
H	-3.897064	-0.381377	2.087901
H	-5.289176	-1.469585	1.895997
H	-5.544641	0.251251	2.287504
H	-0.765133	1.678168	0.212329
H	1.407662	1.580720	0.327165
H	2.732903	-1.158729	-0.250474
H	3.802077	1.662602	0.465372
H	6.457062	-1.276093	-0.603942
H	4.763417	-1.590200	-0.947047
H	6.104022	-1.861005	1.823541
H	5.619096	-3.200148	0.764972
H	4.384326	-2.160443	1.502172
H	7.033946	0.518558	0.962165
H	5.923124	1.880682	1.016366
H	7.257003	0.813293	-1.556529
H	7.733626	2.272384	-0.670824
H	6.148834	2.195585	-1.471466
H	-0.612730	-1.779982	-0.659062
